# Three Dimensional Evaluation of Posterior Pole and Optic Nerve Head in Tilted Disc

**DOI:** 10.1038/s41598-018-19242-z

**Published:** 2018-01-18

**Authors:** Yong Chan Kim, Ji-Sun Moon, Hae-Young Lopilly Park, Chan Kee Park

**Affiliations:** Department of Ophthalmology, College of medicine, Seoul St. Mary’s Hospital, The Catholic University of Korea, Seoul, Korea

## Abstract

For over a century, tilted disc syndrome (TDS) has been defined vaguely. The lack of consensus of the terminology arises from the lack of understanding of the pathogenesis of this condition. Also, myopic discs with temporal crescents or peripapillary atrophy (PPA) are histologically indistinguishable from TDS. Therefore, we examined the morphological background of the extreme ONH appearances such as the myopic tilted disc and the TDS by analyzing the posterior segment of the eye from a three-dimensional (3D) perspective. 107 eyes of 107 subjects were classified into 3 groups with respect to the optic disc torsion degrees: (1) mild torsion (0–30 degrees; 35 eyes) and (2) moderate torsion (30–60 degrees; 35 eyes) and (3) severe torsion (60–90 degrees; 37 eyes). SSOCT images were analyzed in coronal view, which supplements anterior-posterior depth (z axis in Cartesian coordinates). The amount of optic disc torsion was significantly correlated with Disc-DPE angle and Fovea-Disc depth (r = 0.548, P < 0.001 and r = 0.544, P < 0.001). In conclusion, we describe specific types of posterior sclera configuration that corresponds to the increasing degree of optic disc torsion, even in the extreme ONH appearances such as the myopic tilted disc and the TDS. These findings suggest that the optic disc appearance is determined by the configuration of the posterior sclera.

## Introduction

For over a century, tilted disc syndrome (TDS) has been recognized. Designations have included oblique disc, inferior conus, coloboma of the disc, dysverted disc, and wet stone papilla^1^. The lack of consensus of the terminology arises from the lack of understanding of the pathogenesis of this condition^2^. Controversy regarding the nature, etiology, and pathogenesis of TDS still exists. TDS is generally considered a form of congenital coloboma, malclosure of the embroyonic ocular fissure because the direction of tilted disc is within the vicinity of the former embryonic fissure^3^. However, there is no convincing evidence of a link between TDS as a congenital malformation. The reported incidence of TDS is as high as 3.5% in Tanjong Pagar study, whereas the incidence of the coloboma 0.5 to 2.2 cases per 10,000 births^4,5^. In addition, a review of the literature over the past 120 years have only few clearly documented instances on the association of congenitally tilted disc with definite colobomas^2,6–8^. Only a few histopathologic specimens have been reported to show the characteristic features of coloboma in TDS.

Histologically, myopic discs with temporal crescents or peripapillary atrophy (PPA) are indistinguishable from TDS because both show absent or attenuated RPE and choroid. The distinction between TDS and myopic disc is simplified by the direction of the PPA^9^, but confusion exists on how an extremely torted disc with myopia should be categorized^10^. Therefore, the strategy for the differentiation of myopic tilted disc with the TDS should give valuable clinical information on how to approach extreme optic nerve head (ONH) appearances.

The clinical definition of TDS is an abnormality consisting of inferonasal tilting of the optic disc, the presence of an inferior or inferonasal cresent, and an ectasia of the lower fundus or inferior staphyloma^11,12^. Ectasia at inferior half of the globe is rather extraordinary definition of an ONH abnormality because it regards the shape of posterior sclera in describing an ONH appearance^13,14^. If one considers the ONH as a structural unit, when one edge of the disc tilts up, the other must go down. On the basis of this assumption, we hypothesized that the tilted disc to the inferior may be a feature that developed due to down sloping of posterior sclera. Therefore, we examined the morphological background of the extreme ONH appearances by analyzing the posterior segment of the eye from a three dimensional (3D) perspective. We previously introduced a method to identify the posterior pole configuration by assessing the coronal view of swept-source optical coherence tomography (SSOCT), which supplements anterior-posterior depth (z axis in Cartesian coordinates) to the two-dimensional coordinate (xy plane)^15^. The purpose of the present study was to determine the posterior pole shape and its correlation with the ONH appearance in the various ONH appearances such as the myopic tilted disc and the TDS.

## Materials and Methods

This retrospective, observational study was approved by the institutional review board of Seoul St. Mary’s Hospital It followed the tenets of the Declaration of Helsinki. The medical records of 145 subjects who visited the glaucoma clinic of the Seoul Saint Mary’s Hospital between September 2016 and July 2017 were reviewed retrospectively. Informed consent were obtained with every participants. At initial work-up, each subject received an comprehensive eye examination including measurement of best-corrected visual acuity (BCVA), refraction, slit-lamp biomicroscopy, gonioscopy, Goldmann applanation tonometry, dilated stereoscopic examination of the optic disc and fundus, achromatic automated perimetry using the Swedish Interactive Threshold Algorithm standard 24–2 test (Humphrey Visual Field Analyzer; Carl Zeiss Meditec, Inc., Dublin, CA, USA), central corneal thickness by ultrasound pachymetry (Tomey Corporation, Nagoya, Japan), axial length with ocular biometry (IOL Master; Carl Zeiss Meditec, Inc.) and a review of their medical history.

To be included, subjects were required to have 3D volumetric scan of SSOCT (DRIOCT Triton, Topcon corporation, Tokyo, Japan) of which the coronal reconstruction of the scans are available. Eyes had to have an open iridocorneal angle on gonioscopic examination, the corrected visual acuity had to be better than 20/40. Eyes with a history of optic neuropathy other than glaucoma, signs of pathologic myopia including myopic choroidal neovascularization, lacquer crack, or angioid streak were excluded.

TDS and myopic optic disc tilt has vague definition with respect to amount optic disc torsion. To verify characteristics of posterior pole by various ONH appearances, we classified subjects on the basis of optic disc torsion 0 to 90 degrees and subdivided into three groups. Mild torsion represented relatively normal ONH appearances, which measured less than 30 degrees of optic disc torsion. Moderate torsion represented myopic tilted disc appearances, which measured 30 to 60 degrees of optic disc torsion. Severe torsion represented TDS appearances, which measured 60 to 90 degrees of optic disc torsion. The mild torsion group was designated as the control and the other two groups were designated as the significant torsion, which age, axial length, visual field, average RNFL thickness were matched between the two groups.

### Measurement of Optic Disc Torsion

Fundus photographs were acquired using a SSOCT scan, each centered on the macula to the 30 degrees periphery. Optic disc appearances were assessed by 2 independent observers (YCK and HYP) with the Topcon Triton built-in software. The intrinsic caliper was operated manually to draw reference lines on the photograph on a liquid crystal display monitor. The optic disc was defined torted when the axis of the maximum optic disc diameter was not aligned with the vertical meridian^16^, the same definition as that adopted in the Blue Mountains study^17^. The vertical meridian was considered a vertical line 90 degrees from a line connecting the fovea to the center of the disc. A positive torsion value indicated inferotemporal torsion (which is counterclockwise torsion in the right eye format), and a negative value indicated superonasal torsion (which is clockwise torsion in the right eye format). Details have been described previously^18^. Each observer, who was masked regarding the patient clinical information and the posterior pole configuration, classified each eye into 3 categories with respect to amount of optic disc torsion: (1) mild torsion (0–30 degrees) and (2) moderate torsion (30–60 degrees) and (3) severe torsion (60–90 degrees).

### Measurement of Optic Disc Tilt

Optic disc tilt was identified by two different measure, horizontal disc tilt, and vertical disc tilt, respectively. Horizontal and vertical tilt angle was measured using the clinical disc margin as the ONH plane and the imaginary line connecting each Bruch’s membrane margin as the reference plane^19^. Degree-of-tilt was defined as the angle between the reference plane and the ONH plane. Angle measurements were performed by two observers (YCK and HYP) with the software intrinsic angle tool. A positive degree of horizontal tilt indicated tilt towards temporal, and a negative horizontal tilt indicated tilt towards nasal. A positive degree of vertical tilt indicated tilt towards inferior, and a negative vertical tilt indicated tilt towards superior.

### Measurement of Posterior Pole Configuration

The detailed specifications of the SSOCT equipment are as follows. This SSOCT (DRIOCT Triton, Topcon Corporation, Tokyo, Japan) has A-scan repetition rate of 100,000 Hz and uses a light source wavelength laser centered at 1050 nm with 100 nm tuning range. The longer wavelength of the light beam and the smaller signal-to-noise ratio roll off makes a deeper scanning depth of 2.6 mm possible^20^. Coronal images are a reconstruction of three-dimensional volumetric scan, produced by intrinsic software of DRIOCT system. Coronal images yields tissue imaging depth of 2.6 mm, which are composed of 1,000 coronal sections^21^. The software uses three averaged reconstructed en face images along the depth axis followed by slight Gaussian spatial filtering to improve image quality. This averaging along the depth direction was within a depth resolution (8 μm) of the swept source OCT system.

**Figure 1:**
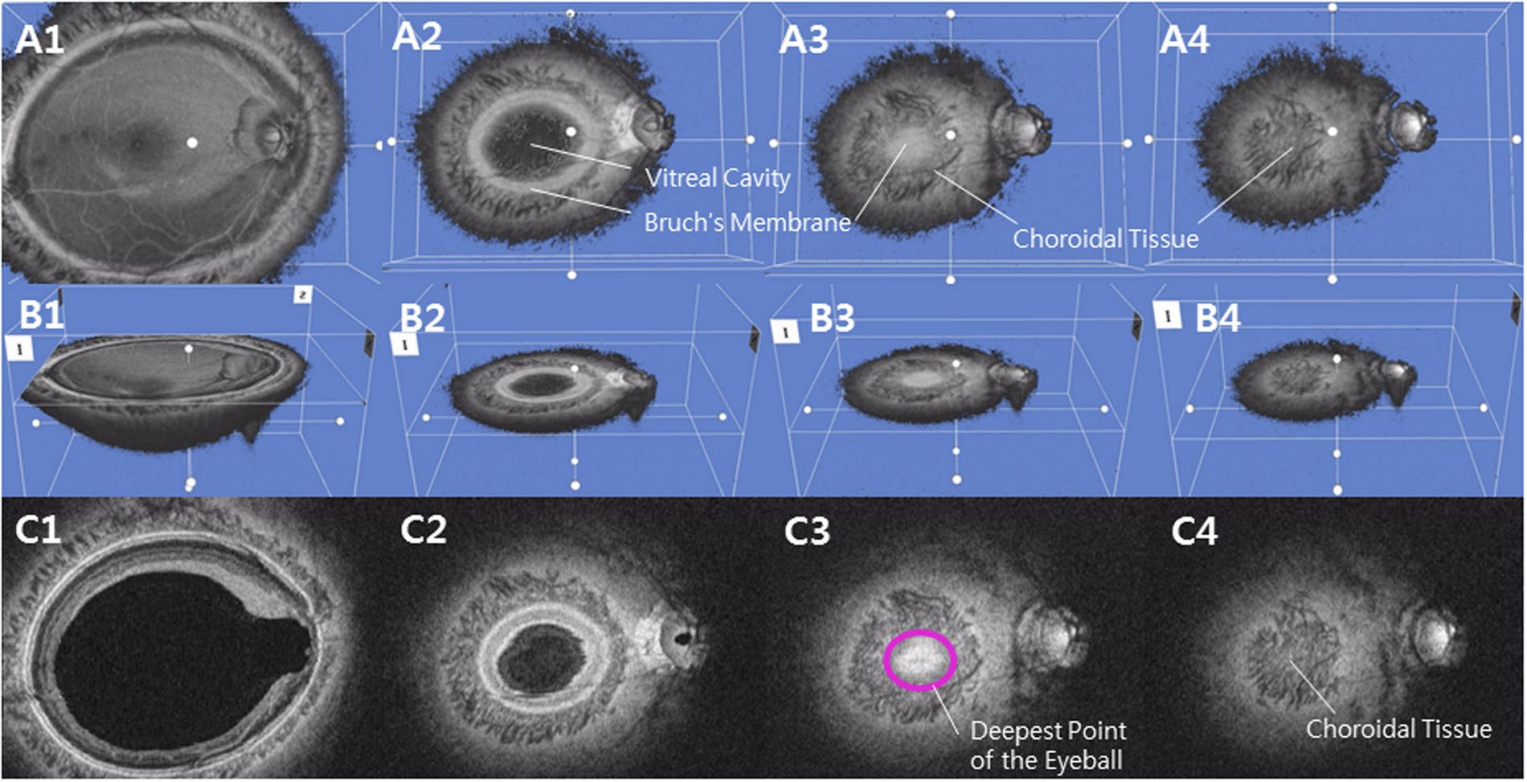
Schematic presentation of the three dimensional assessment of the posterior globe. (**A**) Coronal section image of the three dimensional reconstruction of the posterior globe. The number increases as the section of the posterior pole proceeds posteriorly. A2 represents shape of a posterior pole with vitreal cavity in the center surrounded by hyper-reflective Bruch’s membrane. A3 represents the interface of the Bruch’s membrane that is surrounded by the hypo-reflective choroid. Choroidal tissue appears when the section proceeds the Bruch’s membrane (A4). (**B**) Coronal section image of the three dimensional reconstruction of the posterior globe seen diagonally. The number indicates the identical section as the coronal section in A1 through A4. (**C**) Coronal section image of the three dimensional reconstruction of the posterior globe seen in En face view. The number indicates the identical section as the coronal section in A1 through A4. Deepest point of the eyeball is indicated in a purple circle in C3.

**Figure 2:**
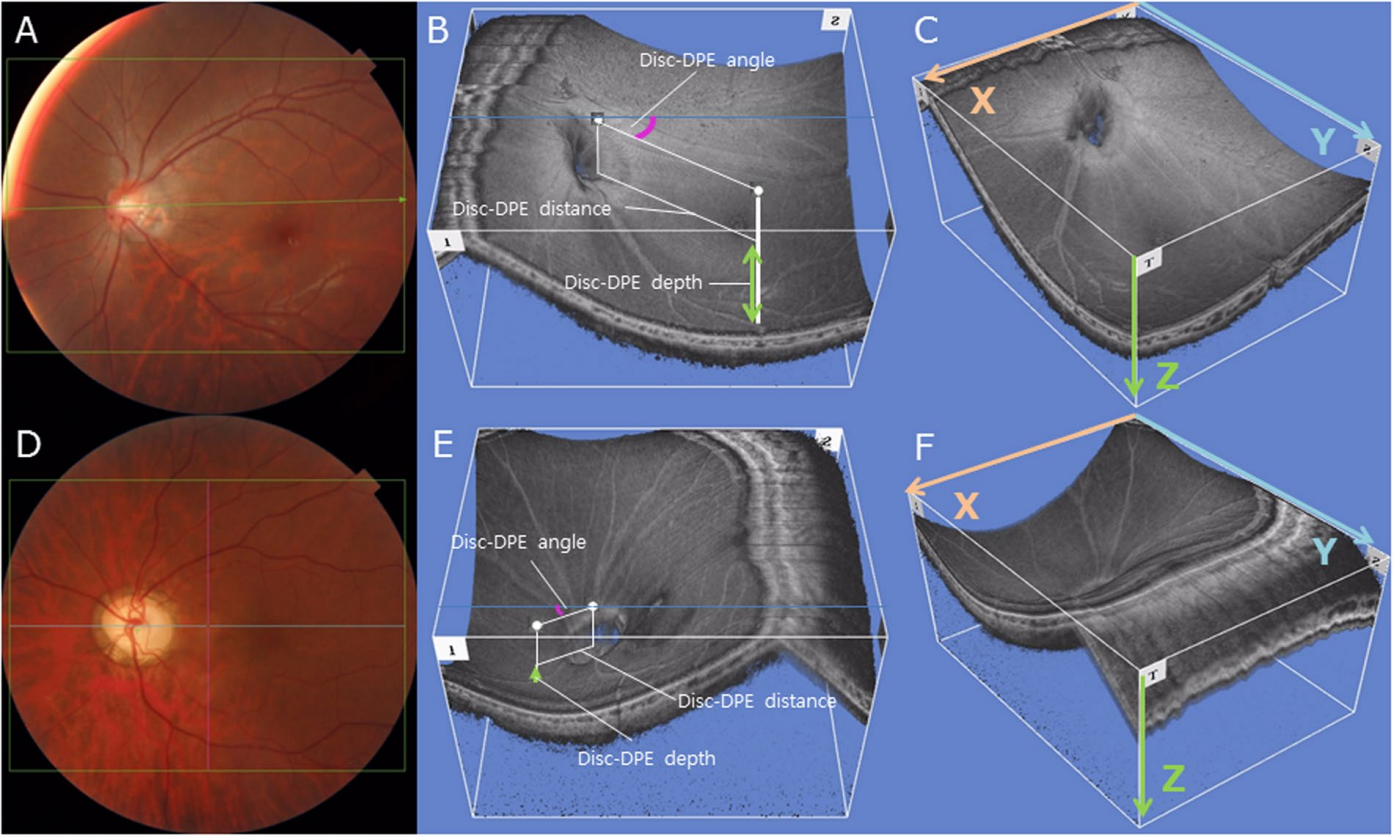
Three dimensional evaluation of the posterior pole in two subjects (**A**,**D**). The Disc-DPE distance and the Disc-DPE angle were parameters measured in the two-dimensional coordinate (xy plane). The Disc-DPE depth was the parameter measured in the anterior-posterior depth (z axis) (**B**,**E**). To the two-dimensional coordinate (xy plane) analysis, this study aim to add the anterior-posterior depth (z axis) in the evaluation of posterior pole (**C**,**F**).

**Figure 3:**
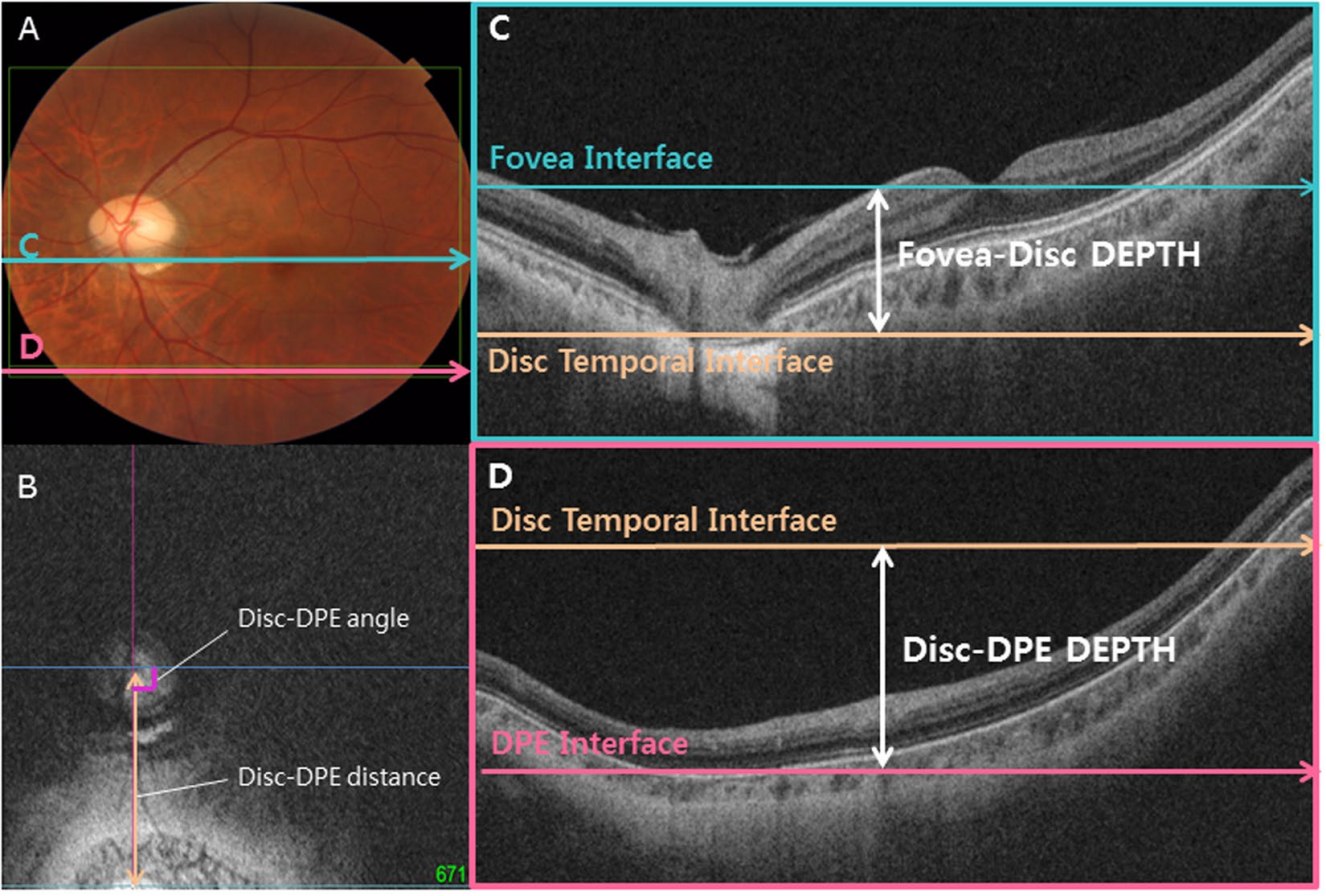
Measurement of the main outcome measures. (**A**) A common fundus photograph of a subject with tilted disc syndrome. (**B**) Coronal section reveals the DPE at the inferior globe. The Disc-DPE distance was defined as the straight-line distance between the center of the optic disc to the center of the DPE measured along the same coronal plane as the DPE. The Disc-DPE angle was defined as the angle between the horizontal meridian crossing the OCT-defined center of the optic disc and the straight line from the OCT-defined center of the optic disc to the DPE. (**C**) A Horizontal section C in section A. The Fovea-Disc depth was the depth between the interface of the fovea and the interface of temporal border of the optic disc. (**D**) A Horizontal section D in section A. The Disc-DPE depth was the depth between the interface of the DPE and the interface of temporal border of the optic disc.

**Figure 4:**
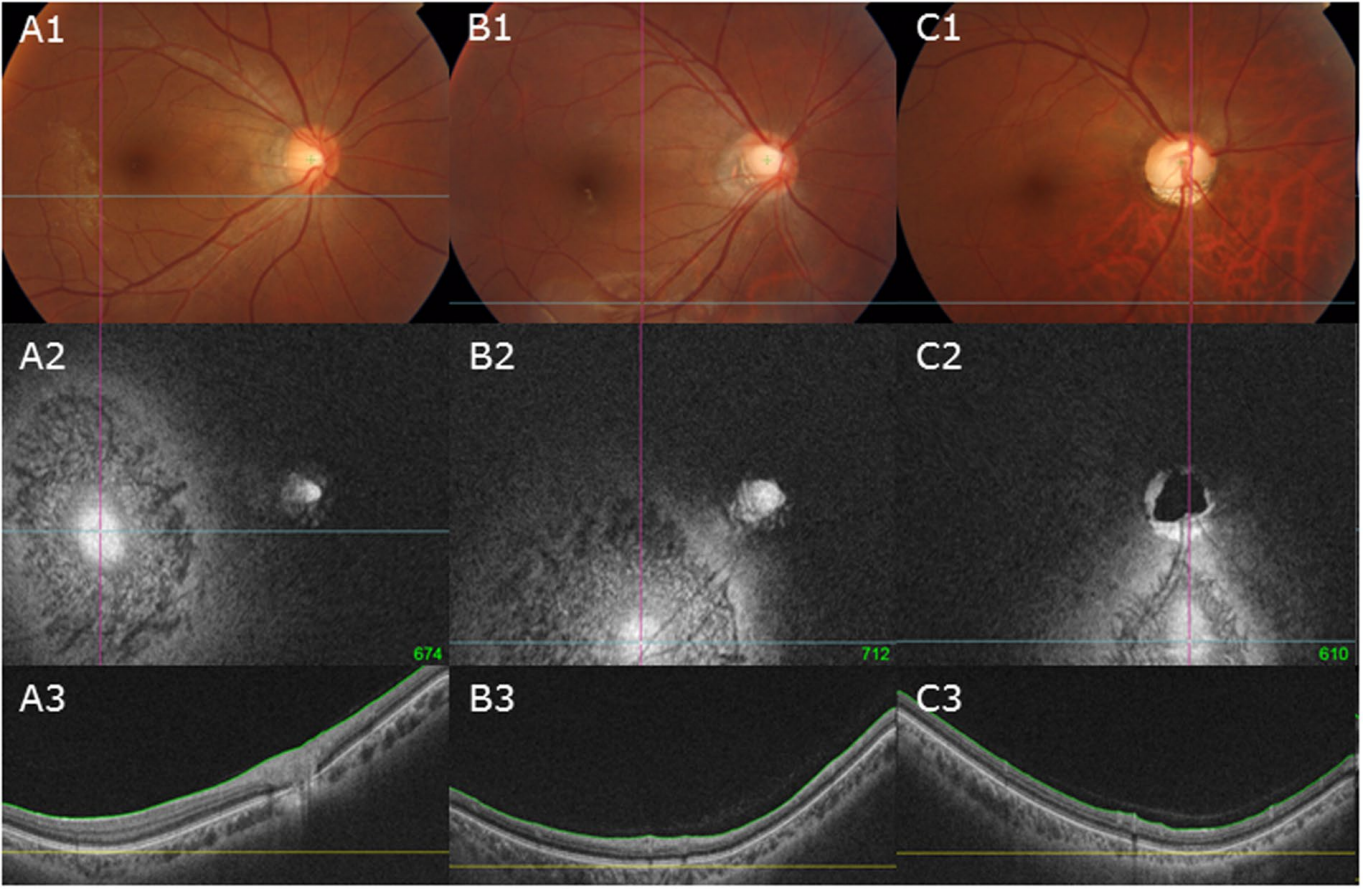
Representation of the classification of the four groups by increasing optic disc torsion (section A1 to C1). (**A**) Mild torsion group represented relatively normal ONH appearances measuring less than 30 degrees of optic disc torsion. (**B**) Moderate torsion group represented myopic tilted disc appearances measuring 30 to 60 degrees of optic disc torsion. (**C**) Severe torsion group represented TDS appearances measuring 60 to 90 degrees of optic disc torsion. Section A2–C2 shows the coronal section and section A3–C3 shows the horizontal section of each subject, respectively.

Coronal image of the posterior segment of the eyeball provides a different perspective from the B-scan cross-sectional imaging, allowing the physician to assess the posterior segment in a three dimensional way (Fig. 1)^22^. From this format, every posterior segment tissues appear as a round figure which contains hypo-reflective vitreous cavity in the middle and hypo-reflective retroorbital fat in the periphery (Fig. 1A,B). The Bruch’s membrane appears as a hyper-reflective round plane and the choroid appears as a round, inhomogeneous hypo-reflective figure with indistinct boundary (Fig. 3A–C). Using the reflectivity difference between different structures, the interface between the different tissues can be located in the consecutive images of coronal section^15^. The interface of the Bruch’s membrane was defined as a hyper-reflective round plane that is surrounded by the hypo-reflective and indistinct choroid (Fig. 3C). The parameter deepest point of the eyeball (DPE) was defined as the deepest (most posterior) interface of the Bruch’s membrane that showed no vitreous cavity in its center, and also with at least amount of the Bruch’s membrane shown inside the choroid tissue (Figs 2C, 3C, 4C). This interface was defined as the DPE of the posterior pole contour (Fig. 3C). Although the outer integument of the eyeball is the sclera, the retinal conformation is better estimated using Bruch’s membrane, rather than the choroid or the sclera, because of their diverse and irregular thickness. So, the measurements of the relative position of the DPE were performed in the coronal plane of the Bruch’s membrane/Choroid interface. Unlike secondary high myopia due to congenital glaucoma, the axial elongation of primary myopia develops primarily in the posterior part of the eyeball^23^. Asymmetric growth of the posterior pole of the eyeball can alter the DPE position from the center of the posterior pole.

Attaining the DPE position in the coronal view is analogous to attaining Cartesian coordinates of the eyeball because it provides the anterior-posterior depth of the eye. DPE measurements in the two-dimensional coordinate (xy plane) were defined as the Disc-DPE distance and the Disc-DPE angle. The anterior-posterior depth (z axis) were defined as the Disc-DPE depth and the Fovea-Disc depth (Fig. 2).

Two observers (YCK and JSM) quantified the DPE location using the caliper function of the Topcon Triton software in a blinded fashion. The SSOCT software indicates the center of the disc as a green cross based on the margin of Bruch’s membrane as a default. The Disc-DPE distance was defined as the straight-line distance between the center of the optic disc to the center of the DPE measured along the same coronal plane as the DPE (Fig. 3B). To quantify the angular position of the DPE relative to the optic disc, the horizontal meridian crossing the OCT-defined center of the optic disc was determined as the reference line. The Disc-DPE angle was defined as the angle between the horizontal meridian crossing the OCT-defined center of the optic disc and the straight line from the OCT-defined center of the optic disc to the DPE measured by the intrinsic caliper of the built-in software (Fig. 3B). Likewise, the Disc-Fovea angle was defined as the angle between the horizontal meridian crossing the OCT-defined center of the disc and the straight line from the OCT-defined center of the optic disc the fovea measured by the intrinsic caliper of the built-in software. The depth between the different posterior pole structures was calculated by the number of coronal sections between the interfaces of the different structures. After determining the depth by counting the number separate images of the coronal section, the depth was converted into micrometers by assuming each coronal section as 2.6 μm in depth. The Disc-DPE depth was the depth between the interface of the DPE and the interface of temporal border of the optic disc (Fig. 3D). Likewise, the Fovea-Disc depth was the depth between the interface of the Fovea and the interface of the optic disc (Fig. 3C).

To compensate for potential errors induced by head tilt or ocular rotation, the subjects were seated at the fundus camera with their chin in the chin rest and forehead against the forehead rest. The subjects’ eyes were aligned with the eye level mark on the forehead rest support by raising or lowering the chin rest. They were instructed to hold their heads in a vertical position throughout the photographic session. Using the eye to be photographed, each patient was instructed to look directly at the internal fixation target in the OCT camera, which was used as a marker for the foveal center. SSOCT has real-time eye tracking that eliminates eye motion and minimizes artifacts by fixating on the fovea on each scan.

### Statistical Analysis

Interobserver reproducibility of measurements of the DPE and ONH parameters were evaluated by calculating intraclass correlation coefficient. The analysis was based on 20 independent series of inter-visit reproducibility conducted twice on different days by two of the authors (YCK and HYP)^24^. Comparisons between the minimal tilt group and the other significant tilt group were performed with chi square and Student *t* tests. To identify a statistically significant trend between the 3 categories with respect to amount of optic disc torsion and the posterior pole configuration parameters, Jonckheere trend test was used. Pearson’s correlation analysis was calculated to assess the relationships of the DPE quantification and the ocular parameters. The level of statistical significance was set at *P* < 0.05.

## Results

A total of 312 subjects who had 3D volumetric scans of SSOCT were included. When both eyes of a patient were eligible for inclusion, only one eye was randomly chosen. Of the 312 eyes of the 312 subjects, we selected more than 30 eyes for each group to follow the normal distribution, respectively. 35 eyes (24.5%) were classified as the mild torsion group, 35 eyes (24.5%) as the moderate torsion group, 37 eyes (25%) as the severe torsion group (Fig. 4).

**Figure 5:**
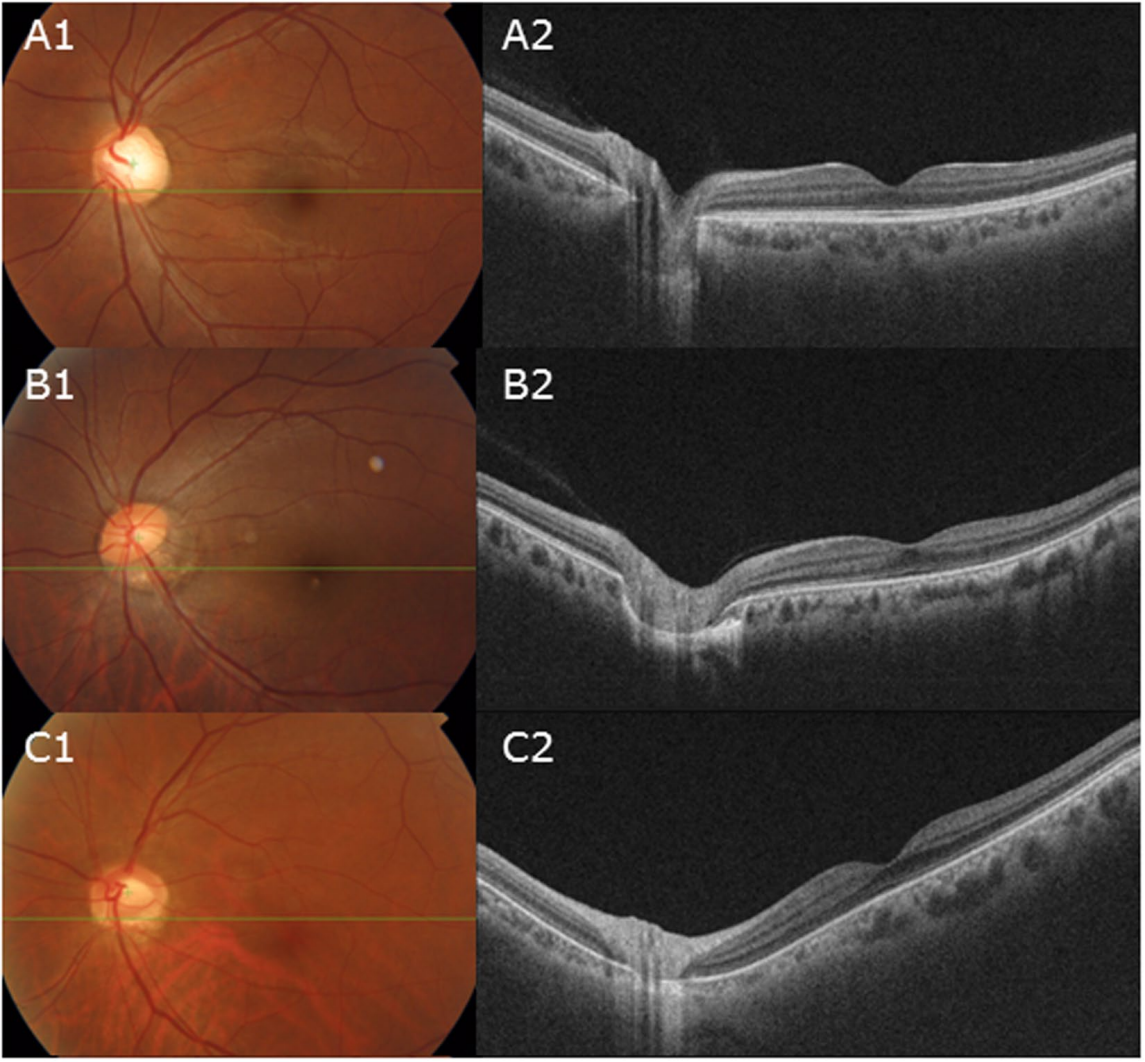
Representation showing the increasing disc torsion degree (section A1 to C1) increased the Fovea-Disc depth (section A2 to C2).

Table 1 summarizes the comparison of baseline characteristics as well as the posterior segment configurations in the three groups. There was excellent interobserver reproducibility in measurement of the Disc-DPE distance, Disc-DPE depth, Fovea-Disc depth and Disc-DPE angle (intraclass correlation coefficient 0.926, 0.995, 0.992 and 0.955, respectively). No statistically significant trend were found between the 3 groups with regard to age, spherical equivalent, axial length, central corneal thickness, visual field MD, average RNFL thickness. However, the measurements of the posterior segment had a statistically significant trend with regard to increasing order of optic disc torsion. The increasing disc torsion degree increased the Disc-DPE angle (Z = 7.414, *P* < 0.001), increased the Fovea-Disc depth (Z = 4.448, *P* < 0.001), and increased the vertical tilt angle (Z = 4.506, *P* < 0.001) (Fig. 5). On the other hand, the increasing disc torsion degree decreased the Disc-DPE distance (Z = −2.709, *P* < 0.001) and decreased horizontal tilt angle (Z = −3.067, *P* < 0.001).

**Table 1:** Baseline characteristics and the posterior pole analysis of the 3 groups*.

	Mild Torsion(*n* = 35)	Moderate Torsion (*n* = 35)	Severe Torsion (*n* = 37)	*JT-*statistic^†^	*Z* ^‡^	*P value* ^§^
Age, years	51.48 ± 14.07	48.88 ± 12.89	54.32 ± 12.68	2,057.0	0.856	0.392
Spherical equivalent, diopter	−3.91 ± 4.43	−5.77 ± 4.06	−4.08 ± 3.53	1,900.0	−0.043	0.966
Axial length, mm	25.65 ± 2.02	26.58 ± 1.26	25.61 ± 1.54	1,849.5	−0.332	0.740
Anterior chamber depth, mm	2.92 ± 0.72	3.14 ± 0.67	3.05 ± 0.59	1,901.5	0.386	0.700
Central corneal thickness, μm	532.90 ± 42.61	532.62 ± 44.78	535.72 ± 40.97	1,897.0	0.362	0.717
Visual field MD, dB	−3.91 ± 4.55	−4.54 ± 6.63	−3.97 ± 5.22	1,909.0	0.009	0.993
Average RNFL thickness, μm	84.25 ± 17.84	78.86 ± 22.25	88.43 ± 18.36	2,164.5	1.471	0.141
Disc-DPE distance, mm	3.99 ± 0.90	4.16 ± 1.42	3.04 ± 1.57	1,434.0	−2.709	**0**.**007**^§^
Disc-DPE depth, mm	0.13 ± 0.13	0.20 ± 0.22	0.12 ± 0.16	1,709.5	−1.133	0.257
Fovea-Disc depth, mm	0.16 ± 0.17	0.28 ± 0.32	0.43 ± 0.22	2,685.0	4.448	**<0**.**001**^§^
Disc–DPE angle (°)	12.89 ± 16.49	41.21 ± 23.59	56.84 ± 28.92	3,203.5	7.414	**<0**.**001**^§^
Disc–fovea angle (°)	7.75 ± 3.91	8.76 ± 3.95	8.94 ± 4.92	2,111.0	1.164	0.244
Disc torsion angle (°)	2.82 ± 5.26	40.20 ± 9.42	73.02 ± 7.48	3,815.0	10.95	**<0**.**001**^§^
Horizontal tilt angle (°)	12.31 ± 8.87	19.31 ± 12.48	5.64 ± 6.81	1,377.0	−3.067	**0**.**002**^§^
Vertical tilt angle (°)	2.74 ± 5.72	18.62 ± 16.07	14.48 ± 11.50	2,675.5	4.506	**<0**.**001**^§^

MD: mean deviation, RNFL: retinal nerve fiber layer, DPE: deepest point of the eyeball.

^*^Data are presented as mean ± standard deviation unless otherwise indicated.

^†^Test statistic value of the Jonkheere-Terpestra test.

^‡^Standardized Test Statistic of the Jonkheere-Terpestra test.

^§^Statistically significant values (*P* < 0.05) are shown in bold.

Table 2 shows the comparisons of the three groups. Age, axial length, visual field MD, average RNFL thickness, Disc-DPE depth showed no statistical difference in any of the three groups. The Disc-DPE distance, Fovea-Disc depth, Disc-DPE angle, Disc torsion angle, horizontal and vertical tilt angle showed significant difference among the groups. Every group had significant difference with each other in the Disc-DPE angle measurement as well as the optic disc torsion and the horizontal tilt angle.

**Table 2:** Multiple comparisons of the 3 groups using the ANOVA and the post-hoc analysis.

	ANOVA^*^P value	(I) Group	(J) Group	*Tukey HSD* ^†^	*Bonferroni* ^‡^
Age, years	0.223				
Axial length, mm	0.121				
Visual field MD, dB	0.869				
Average RNFL thickness	0.120				
Disc-DPE distance, mm	**0**.**001**	Mild	Moderate	0.870	1.000
			Severe	**0**.**008**	**0**.**009**
		Moderate	Severe	**0**.**002**	**0**.**002**
Disc-DPE depth, mm	0.087	Mild	Moderate	0.188	0.243
			Severe	0.954	1.000
		Moderate	Severe	0.099	0.120
Fovea-Disc depth, mm	**<0**.**001**	Mild	Moderate	0.076	0.090
			Severe	**<0**.**001**	**<0**.**001**
		Moderate	Severe	**0**.**042**	**0**.**048**
Disc–DPE angle (°)	**<0**.**001**	Mild	Moderate	**<0**.**001**	**<0**.**001**
			Severe	**<0**.**001**	**<0**.**001**
		Moderate	Severe	**0**.**017**	**0**.**018**
Disc torsion angle (°)	**<0**.**001**	Mild	Moderate	**<0**.**001**	**<0**.**001**
			Severe	**<0**.**001**	**<0**.**001**
		Moderate	Severe	**<0**.**001**	**<0**.**001**
Horizontal tilt angle (°)	**<0**.**001**	Mild	Moderate	**0**.**008**	**0**.**009**
			Severe	**0**.**011**	**0**.**012**
		Moderate	Severe	**<0**.**001**	**<0**.**001**
Vertical tilt angle (°)	**<0**.**001**	Mild	Moderate	**<0**.**001**	**<0**.**001**
			Severe	**<0**.**001**	**<0**.**001**
		Moderate	Severe	0.305	0.426

^*^Analysis of variance for the difference analysis among groups.

^†^Tukey’s honest significance test for post-hoc anaylsis.

^‡^Bonferroni method for post-hoc anaylsis.

**Figure 6:**
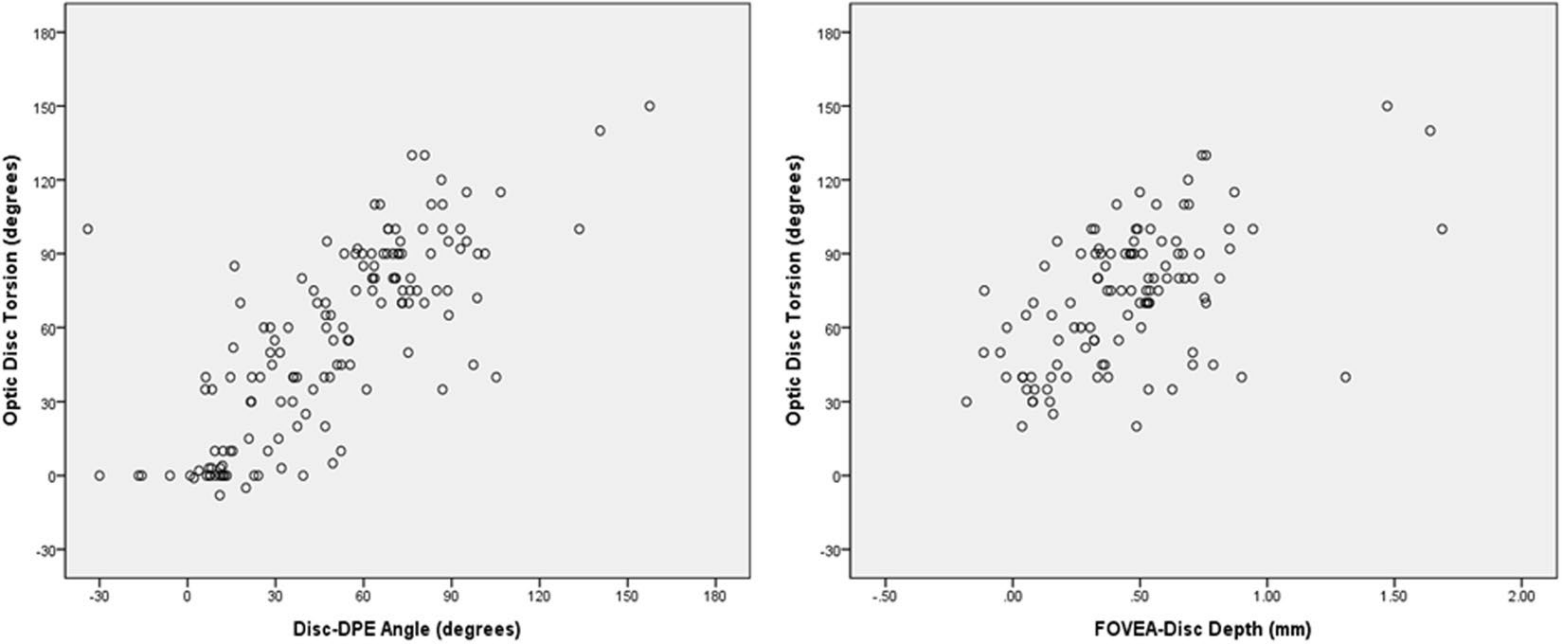
Scatter plot showing the degree of optic disc torsion and the Disc-DPE angle were significantly correlated (*r* = 0.548 and *P* < 0.001) and the Fovea-Disc depth were also significantly correlated with the degree of optic disc torsion (*r* = 0.544 and *P* < 0.001).

Correlation analysis of all subjects revealed that optic disc torsion was significantly associated with increasing age, Fovea-Disc depth, and Disc-DPE angle (*P* = 0.024, *P* < 0.001, and *P* < 0.001, respectively), and decreasing axial length, Disc-DPE distance, and Disc-DPE depth (*P* = 0.001, *P* < 0.001, and *P* = 0.004, respectively) (Table 3). The degree of optic disc torsion and the Disc-DPE angle were significantly correlated (*r* = 0.548 and *P* < 0.001) (Fig. 5). Fovea-Disc depth was also significantly correlated with the degree of optic disc torsion (*r* = 0.544 and *P* < 0.001) (Fig. 6).

**Table 3:** The correlation analysis on the posterior pole configuration and the optic nerve head.

Variables	Optic Disc Torsion		Disc-DPE Angle		Disc-DPE Distance		Disc-DPE Depth		Fovea-Disc Depth	
	*R*	*P* Value^*^	*R*	*P* Value^*^	*R*	*P* Value^*^	*R*	*P* Value^*^	*R*	*P* Value^*^
Age, years	0.216	0.024	0.191	0.049	−0.403	<0.001	−0.291	0.002	0.210	0.028
Axial length, mm	−0.306	0.001	−0.137	0.153	0.312	0.001	0.261	0.006	−0.018	0.851
Visual Field MD, dB	−0.017	0.861	−0.039	0.683	−0.080	0.408	−0.131	0.172	−0.038	0.690
Average RNFL thickness, μm	0.086	0.373	0.139	0.149	−0.205	0.031	−0.199	0.037	0.013	0.892
Disc-DPE distance, mm	−0.432	<0.001	−0.403	<0.001			0.792	<0.001	−0.578	<0.001^†^
Disc-DPE depth, mm	−0.273	0.004	−0.231	0.015	0.792	<0.001			−0.468	<0.001^†^
Fovea-Disc depth, mm	0.544	<0.001	0.653	<0.001	−0.578	<0.001	−0.468	<0.001		
Disc–DPE angle (°)	0.548	<0.001			−0.403	<0.001	−0.231	0.015	0.653	<0.001
Disc–fovea angle (°)	0.072	0.457	0.309	0.001	−0.050	0.602	0.075	0.434	0.271	0.004
Disc torsion angle (°)			0.548	<0.001	−0.432	<0.001	−0.273	0.004	0.544	<0.001
Ovality index	−0.071	0.460	0.030	0.758	0.259	0.006	0.349	<0.001	0.039	0.687
Horizontal tilt angle (°)	−0.497	<0.001	−0.183	0.056	0.212	0.026	0.086	0.373	−0.066	0.494
Vertical tilt angle (°)	−0.048	0.619	0.080	0.409	0.092	0.338	0.066	0.495	0.208	0.029

^*^Pearson’s correlation analysis.

## Discussion

We describe specific types of posterior sclera configuration that corresponds to the increasing degree of optic disc torsion. Even in the extreme ONH appearances such as the TDS and myopic tilted disc, the optic disc appears torted in the direction of the steepest down sloping of posterior sclera. Furthermore, with increasing degree of disc torsion, the ONH region protrudes towards the posterior. To the best of our knowledge, this is first documentation identifying the morphological background of the optic disc torsion in varied degrees.

The TDS is defined inconsistently in many aspects. The TDS is considered to be a congenital malformation that is a consequence of malclosure of the embryonic ocular fissure^10^. However, it is very unconvincing to define a congenital malformation if it lacks histologic evidence^6,7^. Our data demonstrates that the TDS may be an appearance of normal ONH that is tilted towards the inferior. The association of the optic disc torsion with the DPE at varied degrees suggests that disc shape corresponds well with the tilting towards the location of deepest point. If one considers the ONH as a structural unit, when one edge of the disc tilts up, the other must go down. The tilted disc to the DPE may be a feature that developed due to down sloping of posterior sclera, wherever the deepest point locates. With the incidence of the TDS as high as 3.5%, a perspective that it is a congenital malformation needs to be reconsidered^5,12^.

Our observation also has implications for differentiating the myopic tilted disc to the TDS. So far, the two are vaguely differentiated with the direction of the PPA, without specific definition of the reference point. Our data suggest that the myopic tilted disc and the TDS have the same principle of determining the ONH appearance. As shown in Table 1 and Fig. 3, there is a consistent trend of the posterior pole configuration as optic disc torsion increases. The myopic tilted disc group (moderate torsion) and the TDS group (severe torsion) had no statistical difference with regard to posterior pole configuration. These findings are supported by the histologic findings of the myopic tilted disc and the TDS that shows insignificant distinction^2,25–27^. Thus, our findings suggest that the myopic tilted disc and the TDS are a similar type of ONH appearance with similar shape of posterior segment of the eyeball.

Based on the foregoing, we posit a specific model of the posterior pole by the amount of optic disc torsion. An eye with mild optic disc torsion would have the location of the DPE near the fovea in the XY plane, and a similar depth with the optic disc temporal border. An eye with myopic tilted disc would have a longer axial length and with the DPE lower than the fovea in the XY plane, and deeper DPE depth than the eye with mild disc torsion. An eye with tilted disc syndrome would have the DPE directly below the optic disc in the XY plane with the ONH region protruding to the posterior. An eye with situs inversus appearance would have the DPE nasal to the disc, with the ONH region protruded most to the posterior. The amount of optic disc tilting measured with disc ovality index suggests that the optic disc tilt is predicted with the Disc-DPE distance and the Disc-DPE depth, not with the amount of optic disc torsion. This theoretical prediction may provide a speculation of normal ONH configuration for each individual. Analyzing the eyes that do not fit with this speculation may provide an additional explanation for the ONH deformation in glaucoma.

**Figure 7:**
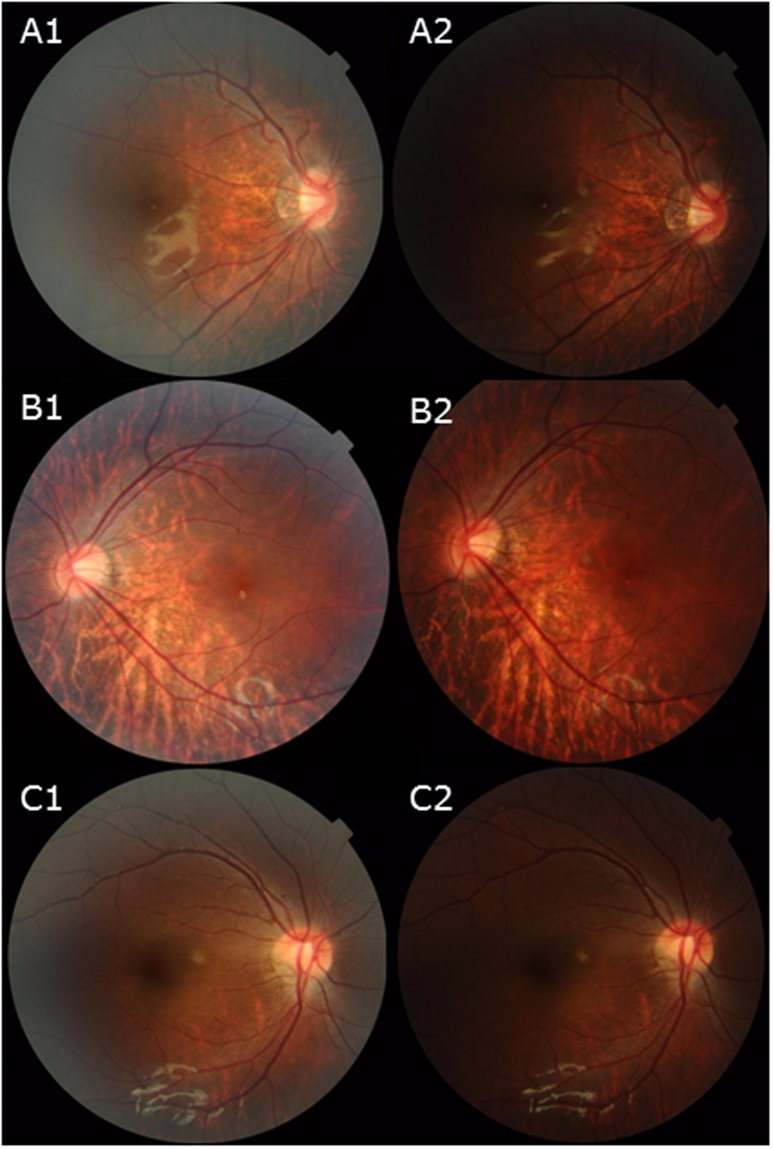
A serial follow up of disc photographs of children. (**A1–2)**. Disc photographs of a 3 year old male subject who was followed-up for 2 years. (**B1–2)**. Disc photographs of a 5 year old female subject who was followed-up for a year. (**C1–2)**. Disc photographs of an 8 year old female subject who was followed-up for 4 years. No specific change of the ONH was evident in all subjects.

Although we suggest a trend based on the degree of optic disc torsion, our findings do not suggest a progressive torsion of the optic disc. No extensive study of the development of the TDS has been reported. However, we have found a child as early as 20 months of age with severely torted disc. Unfortunately, the child was not eligible for the SSOCT scan, but showed consistent torsion throughout 3 years of follow-up (Fig. 7). For a child that young to develop a stable and severe torsion, we assume that the initial alteration of the posterior sclera should be located towards the inferior. Our hypothesis posits that the optic disc torsion is not a progressive change of the ONH, but rather is a predictable element of ONH based on the DPE. However, a misalignment of optic disc torsion with the Disc-DPE angle is present. In the axial elongation process, scleral stretching is present. If an excessive mechanical stress is concentrated in an area of the posterior pole, distortion of the posterior pole configuration can occur. This distortion may cause an irregularity of the posterior sclera, thus triggering misalignment. This theoretical framework may provide an additional explanation of ONH morphology and will be further studied.

Shinohara *et al*.^28^ recently examined the tilted disc syndrome using SSOCT and three-dimensional magnetic resonance imaging (3D MRI). They reported some characteristics consistent with our finding. The authors described that the inferior staphyloma may induce the characteristic optic disc findings of TDS, but did not analyze the ONH elaborately to draw the conclusion that the optic disc torsion, regardless of its extent, can be explained by the general principle of background posterior pole configuration. The authors also presented 3D MRI images of the posterior pole from the sagittal and rear views, and clearly demonstrated the posterior protrusion of the ONH region, which is consistent with our internal analysis of the eye. This finding can be further discussed with our data that the increasing degree of disc torsion decreases the axial length measured at the fovea. When the normal eyeball grows axially, the temporal sclera moves back and becomes flattened^29^. Our data suggests in cases with the extreme disc torsion, unlike normal eyeball elongation, the ONH region protrudes posteriorly. Because measuring the axial length uses the ultrasound at the fovea, axial length could be underestimated in regards to the severe alterations of the posterior pole, mainly in the ONH region.

The study has several limitations. First, we were unable to demonstrate conclusively that the TDS develops congenitally. Although a few children with a significantly torted disc were assessed, we were unable to scan any infants with an OCT because of the cooperation and head size issues. TDS at the time of birth has not well reported. So, the hypothesis that the TDS develops with the development of an altered posterior sclera can be made with assumption of causality. Second, the DPE position is stable only when the subject is fixated at the scanning light. Nonetheless, any equipment that analyzes the fundus assumes that the subject is fixated at the scanning light. Even though DPE needs to be verified, just like any other structures of the fundus photograph, DPE position is reproducible as long as the proper fixation is achieved. Third, counting the number of coronal sections to estimate tissue depth is not an accurate measurement, but only an estimation, to compare between different DPE of individuals. Yet, as of now, there is no way to measure the exact depth of the posterior pole *in vivo*.

In conclusion, we document specific types of posterior sclera configuration that corresponds to an increasing degree of optic disc torsion, even in extreme ONH appearances like the myopic tilted disc and TDS. With increasing degree of disc torsion, the location of the DPE corresponds with the torsion and the ONH region protrudes towards the posterior. These data suggest that the configuration of the posterior sclera determines optic disc appearance irrespective of how the ONH appears.
